# Nonlinear dynamical model based control of *in vitro* hippocampal output

**DOI:** 10.3389/fncir.2013.00020

**Published:** 2013-02-20

**Authors:** Min-Chi Hsiao, Dong Song, Theodore W. Berger

**Affiliations:** ^1^Department of Biomedical Engineering, University of Southern CaliforniaLos Angeles, CA, USA; ^2^Department of Biomedical Engineering, Center for Neural Engineering, University of Southern CaliforniaLos Angeles, CA, USA; ^3^Department of Biomedical Engineering, Program in Neuroscience, and Center for Neural Engineering, University of Southern CaliforniaAngeles, CA, USA

**Keywords:** neural prosthesis, Volterra kernel, inverse control, trajectory model, hippocampus

## Abstract

This paper describes a modeling-control paradigm to control the hippocampal output (CA1 response) for the development of hippocampal prostheses. In order to bypass a damaged hippocampal region (e.g., CA3), downstream hippocampal signal (e.g., CA1 responses) needs to be reinstated based on the upstream hippocampal signal (e.g., dentate gyrus responses) via appropriate stimulations to the downstream (CA1) region. In this approach, we optimize the stimulation signal to CA1 by using a predictive DG-CA1 nonlinear model (i.e., DG-CA1 trajectory model) and an inversion of the CA1 input–output model (i.e., inverse CA1 plant model). The desired CA1 responses are first predicted by the DG-CA1 trajectory model and then used to derive the optimal stimulation intensity through the inverse CA1 plant model. Laguerre-Volterra kernel models for random-interval, graded-input, contemporaneous-graded-output system are formulated and applied to build the DG-CA1 trajectory model and the CA1 plant model. The inverse CA1 plant model to transform desired output to input stimulation is derived from the CA1 plant model. We validate this paradigm with rat hippocampal slice preparations. Results show that the CA1 responses evoked by the optimal stimulations accurately replicate the CA1 responses recorded in the hippocampal slice with intact trisynaptic pathway.

## Introduction

A neural prosthesis is a prosthetic device that interfaces with the nervous system to improve or restore impaired neural function (Berger et al., 1994; Schwartz, 2004; Patil and Turner, 2008). The neuroprosthetic technology has been advancing rapidly (Bernotas et al., 1986; Creasey et al., 2004; Mayberg et al., 2005; Hochberg et al., 2006; Allison et al., 2007; Stacey and Litt, 2008). Neural prostheses can be categorized according to the directions of the signal communication between the device and the nervous system (Turner et al., 2005; Song et al., 2007). The first category of neural prostheses attempts to decode neural signals and then to activate an external object. An example would be the neuroprobes decoding motor cortex signals to control a robotic arm (Donoghue, 2002; Nicolelis, 2003; Taylor et al., 2003). The second kind of neural prostheses encodes external sensory stimuli and intends to activate the nervous system. Examples are cochlear implants and artificial retinas (Middlebrooks et al., 2005; Weiland et al., 2005). The third kind of neural prostheses, which forms a bi-directional closed-loop system with the nervous system, receives incoming neural signals from one nervous region and sends its output to activate another nervous system region (Berger et al., 2001, 2011). For the neural prosthesis that involves stimulation to the nervous system, the output system responses could be influenced by the stimulation parameters such as location, intensity, and frequency. Because the signal transformation in the nervous system is nonlinear, it is also important to consider the nonlinearity between stimulation patterns and the output responses. Without considering this nonlinear relationship, large deviations between the device-evoked responses and the desired responses are expected. In practice, such deviations can be mitigated by tuning the stimulation parameters (Lauer et al., 2000; O'Suilleabhain et al., 2003; McIntyre et al., 2004; Tellez-Zenteno et al., 2006; Rupp and Gerner, 2007; Albert et al., 2009; McLachlan et al., 2010). This optimization procedure is typically performed manually and empirically, e.g., assuming a static and linear relation between the stimulation pattern and the desired responses, and then searching for the optimal ratio between the stimulation intensity and the outcome responses via a trial-and-error procedure. To formally solve this important problem, one needs to develop a rigorous stimulation paradigm that takes the (nonlinear dynamical) relationship between stimulation signals and system responses into account (Liu and Oweiss, 2010; Liu et al., 2011).

We are in the process of developing a neural prosthesis to restore the long-term memory formation function of the hippocampus that is lost in Alzheimer's disease, stroke, epilepsy, or other neurological disorders. Our concept of such a prosthetic device is a biomimetic model of the input–output nonlinear dynamics of the hippocampus—a model that captures how hippocampal circuitry re-encodes, or transforms, incoming spatio-temporal patterns of neural activity (i.e., short-term memories) into outgoing spatio-temporal patterns of neural activity (i.e., long-term memories) (Squire, 1992; Berger et al., 2001, 2005; Burgess et al., 2002). We have shown in rodents, both *in vitro* (Chan et al., 2004; Hsiao et al., 2006) and *in vivo* (Song et al., 2007, 2009; Berger et al., 2011, 2012), that a nonlinear hippocampal model is capable of predicting accurately the output signals based on the ongoing input signals in the hippocampus. In this study, we extend this concept by developing a rigorous stimulation paradigm with control theory, and then implementing it rat hippocampal slices.

**Figure 1:**
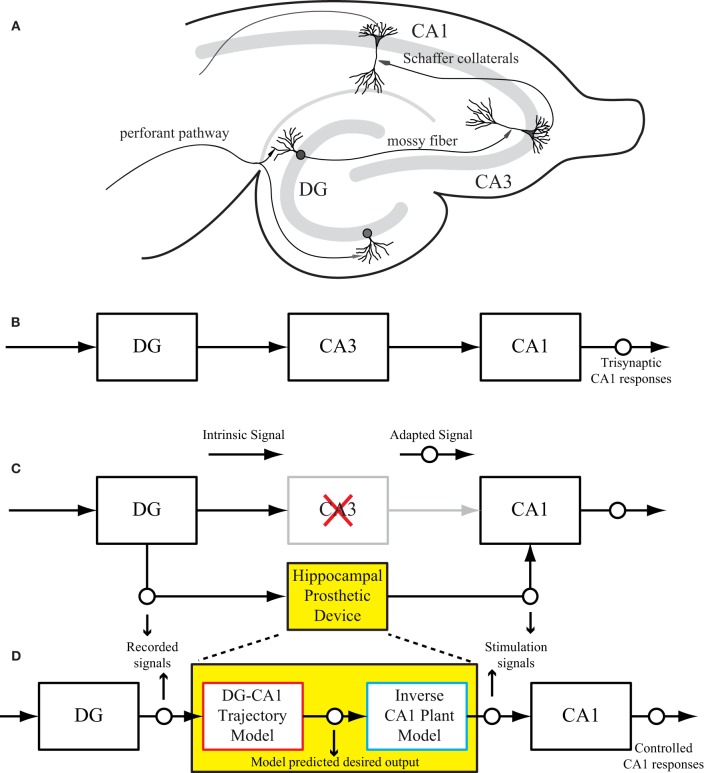
**(A)** A rat hippocampal slice and its major intrinsic pathways. The input signals from perforant path fibers excite dentate granule cells. Dentate output, in turn, excites CA3 pyramidal cells through mossy fibers. Output from CA3 is transmitted to CA1 pyramidal cells through Schaffer collaterals. This so-called “trisynaptic pathway” is the principal network involved in hippocampal neuronal information processing. **(B)** A block diagram showing the trisynaptic pathway in a hippocampal slice. **(C)** A schematic diagram of a hippocampal prosthesis model functionally replacing the original pathway, where CA3 is damaged, so the signal transmission cannot be completed. This bi-directional prosthetic device receives incoming neural signals from one hippocampal region (DG) and sends its output to stimulate another hippocampal region (CA1). **(D)** The proposed modeling-control paradigm to optimize the stimulation patterns. In this framework, the desired CA1 output is first predicted with the DG signal by the trajectory model, and then converted to the desired stimulation patterns through the inverse model. The desired stimulation patterns then drives the output system (CA1) to the desired output responses.

The intrinsic circuitry of the hippocampus consists of three major subregions: dentate gyrus (DG), CA3, and CA1 as shown in Figure 1A. This trisynaptic circuit can be maintained in a transverse slice preparation (Andersen et al., 1969, 2000; Amaral and Witter, 1989). The signal transformations in all three regions are highly nonlinear and dynamical (Berger et al., 1988; Sclabassi et al., 1988; Bartesaghi et al., 2006). From an engineering perspective, the hippocampal circuit can be viewed as a cascade of input–output transfer functions between the DG, CA3, and CA1 subregions (Figure 1B). In the context of extracellular recording as in this study, the evoked field potentials in each subsystem are measured as input–output signals. For example, the CA3 response (field excitatory postsynaptic potentials amplitude, fEPSP) can be used as the input signal to CA1, and the CA1 response can be considered as the final system output. A schematic diagram of such a hippocampal prosthesis is shown in Figure 1C, where CA3 is damaged, thus the signal transmission from DG to CA1 cannot be completed. In the replacement scenario, the prosthesis model processes the DG signals and generates optimal stimulations to elicited desired output response in the CA1 region.

The successful implementation of such a device depends on three sequential components. First, the device must capture incoming neuronal signals reliably from the input region. Second, it must mimic the damaged system precisely through a computational model. Finally, the device should reproduce the desired responses in the output region through electrical stimulation. Thus, through bi-directional communication with the brain, the prosthetic device could essentially bypass the damaged region and substitute the lost function.

This paper describes the procedure of deriving optimal stimulation patterns using an inverse control concept (Houk, 1988; Widrow and Walach, 1996; Camacho and Bordons, 2003; Normann, 2007). The “trajectory model” is a model that predicts the desired output response based on the input patterns. This model can be developed using available knowledge or built directly from experimental input–output data. The stimulation-response properties of the output system is described as the “plant model.” The “inverse plant model” describes a system whose transfer function is the inverse transformation of the plant model (Widrow et al., 1993; Widrow and Plett, 1997; Karniel et al., 2001). This inverse transformation can be determined once the input–output transformation of the plant model is fully explored. Once these three models (i.e., trajectory, plant, and inverse plant models) are built, the signals flow like what is shown in Figure 1D. Signals recorded from the DG (input) system pass through the trajectory model to predict the desired output. The inverse plant model is then used to derive the desired stimulation amplitudes from desired output. Finally, the CA1 (output) region generates the controlled output responses. Results show that the strategy described in this paper is able to control CA1 output activities (shown in Figure 1D as “Controlled CA1 responses”) to replicate the CA1 activities recorded from the hippocampal slice with intact trisynaptic pathway (shown in Figure 1B as “Trisynaptic CA1 responses”).

## Materials and methods

The proposed modeling-control paradigm was verified using an *in vitro* rat hippocampal slice preparation. Section “Experimental Procedures” provides an explanation of the methodology used to prepare the hippocampal slices, and the description of our electrophysiology experimental setup. Section “Modeling-Control Paradigm Implementation and Data Collection” describes the estimations and validations of the trajectory model, the plant model and the inverse plant model, and the associated data collection and analysis procedures. The overall experimental protocol is described in section “Modeling-Control Framework Experiment Protocol.”

### Experimental procedures

#### Acute hippocampal slice preparation

Hippocampal slices from 8 to 10-week-old male Sprague-Dawley rats (250–300 gm) were prepared. The animals were first anesthetized with halothane (Halocarbon Laboratory, USA) and then decapitated. Their skulls were rapidly removed and the brain was carefully extracted. Hippocampi were separated from the cortices in an iced sucrose buffer solution (Sucrose 206 mM; KCl 2.8 mM; NaH_2_PO_4_ 1.25 mM; NaHCO_3_ 26 mM; Glucose 10 mM; MgSO_4_ 2 mM; Ascorbic Acid 2 mM). Hippocampal slices 400 micrometers thick were sliced transversely from the ventral hippocampi using a vibratome (Leica VT1000S, Germany). The slices were incubated for at least 1 h in 2 mM MgSO_4_ artificial cerebral spinal fluid (aCSF) at room temperature, to equilibrate. During each electrophysiological recording session, one slice at a time was transferred to the planar multielectrode array. The array attached with a circular plastic chamber and perfused with normal aCSF (NaCl 128 mM; KCl 2.5 mM; NaH_2_PO_4_ 1.25 mM; NaHCO_3_ 26 mM; Glucose 10 mM; MgSO_4_ 1 mM; Ascorbic Acid 2 mM; CaCl_2_ 2 mM) maintained at room temperature (24~26°C). In the recording chamber, each slice was held down by a metallic ring with nylon mesh attached to it. The positioning of the slice was accomplished by manipulating the ring with a small brush. All the solutions were bubbled with 95% O_2_ and 5% CO_2_ mixed gas. The protocol described above was approved by the Department of Animal Resources and Institutional Animal Care and Use Committee at the University of Southern California.

#### Electrophysiological recording setup and procedures

**Figure 2:**
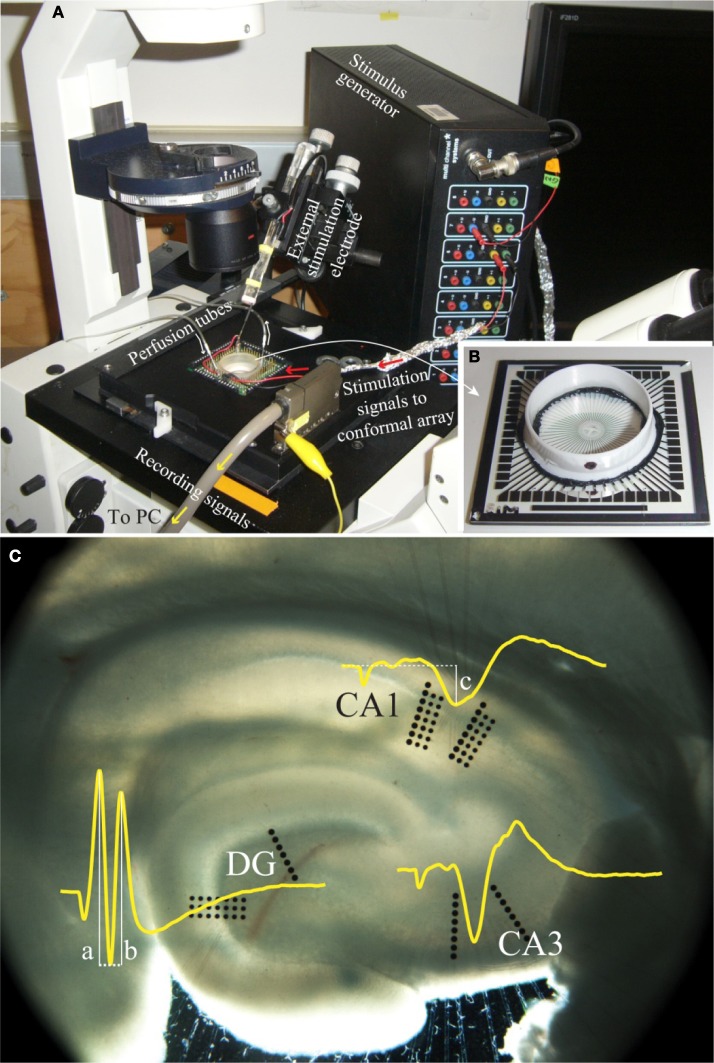
**A photo of the electrophysiological recording system. (A)** The MEA60 system and **(B)** the conformal planar MEA (Gholmieh et al., 2006; Taketani and Baurdy, 2006). **(C)** A photomicrograph of a hippocampal slice on the conformal MEA. The set alignment of this array is according to rat hippocampal cytoarchitecture covering major subregions of DG, CA3, and CA1. The waveforms represent the trisynaptic response of the hippocampal slice recorded in each region. The white lines indicate the amplitude measurement of DG population spike amplitude and CA1 fEPSP amplitude (see section “FARIT-Induced Trisynaptic Data Collection and Analysis”).

Electrophysiology data were collected through an extracellular recording technique using an MEA60 system (Multi Channel Systems, Germany), as seen in Figure 2. This system consisted of pre-amplifiers (1200× gain), a data acquisition device (MC_Card), and an 8-channel stimulus generator (STG1008), all operated using software provided by Multi Channel Systems (MC_Rack V3.2.0 and MC_Stimulus V2.0.6). A conformal 60-channel planar multielectrode array was made specifically for this study. The geometry of this conformal array was designed to match the cytoarchitecture of the hippocampus slices (Figure 2C) and was platinum based. Details in fabrication and the arrangement of the array can be found in Gholmieh et al. (2006) and Taketani and Baurdy (2006). Collected data were sampled at a frequency of 10 kHz per channel and were recorded using MC_Rack. The MEA60 system was assembled over an inverted microscope (Leica DM-IRB, Germany). In each experiment, the position of the slice on the MEA was captured by a digital image capture system (Diagnostic Instruments, Spot RT Digital Camera, USA) with SPOT (V4.6.4.3) software and Adobe Photoshop (Adobe V7.0, USA).

#### Stimulation and data collection procedures

In this study, biphasic currents with a 100 μs duration in each phase were applied to all stimulation patterns. Different stimulation trains were programmed in MC_Stimulus and used to study the nonlinear properties of different regions. There was a 5–7 min waiting period between each stimulus train. The evoked neural responses were simultaneously recorded from different regions. The channels were first selected based on the placement of the recording electrodes on the cytoarchitecture of the slices (i.e., DG channels must be in the DG region, CA3 channels must be in the CA3 region, etc). Among those channels, the channels with the largest response (dendritic population spike or EPSP) amplitudes are further selected and analyzed. The main purpose of this procedure is to find the most representative responses for each region, channels with small responses or inappropriate placements are not analyzed because their recordings may reflect a non-cell-body placement or a mixture of activities from multiple regions.

### Modeling-control paradigm implementation and data collection

#### DG-CA1 trajectory model implementation

***FARIT-induced trisynaptic data collection and analysis.*** An external bipolar electrode of twisted Nichrome wires was used to elicit the trisynaptic response. Paired-pulse or quadruplet-pulse electrical stimulation was applied to the perforant pathway of each slice using the external electrode to generate electrophysiological responses throughout the trisynaptic pathway (evoked field potentials in DG, CA3, and CA1, as seen in Figure 2C). When the full trisynaptic response was observed, we stimulated the slice with a series of fixed-amplitude, random inter-impulse-interval trains (FARITs). Four 300-pulse Poisson distributed FARITs of a fixed current intensity (biphasic, 150–300 μA) were delivered to the perforant path (1200 impulses; range of intervals: 2 ms to 5 s; mean frequency: 2 Hz). Response amplitudes from selected channels in DG and CA1 regions were analyzed to build the DG-CA1 trajectory model. The neuron response measurement in DG was population spike amplitude, the amplitude was calculated by averaging the distance between the negative peak and the first positive peak (measure “a” in Figure 2C) and the distance between the negative peak and the second positive peak (measure “b” in Figure 2C) (Houk, 1988). To measure responses in the CA1 regions, the field potential amplitude was defined as the negative peak of the waveform (measure “c” in Figure 2C).

***Trajectory model configuration.*** A single-input, single-output discrete model was derived from Volterra series as expressed below (Marmarelis and Orme, 1993; Marmarelis, 2004):

(1) $$\begin{array}{c} \begin{array}{l} y(n)=k_{0}+\sum_{m=0}^{M}k_{1}(m)x(n-m)\\ \;+\sum_{m_{1}=0}^{M}\sum_{m_{2}=0}^{M}k_{2}(m_{1},m_{2})x(n-m_{1})x(n-m_{2})+\ldots \end{array} \end{array}$$

The zeroth order kernel *k*_0_ is the value of output *y* when the input is absent. First order kernels *k*_1_ describe the relationship between each single input *x*(*n* − *m*) and output *y*. Second order kernels *k*_2_ describe the relationship between the output *y* and each unique pair of input *x*(*n* − *m*_1_), *x*(*n* − *m*_2_). The term *n* represents time of occurrence of the present impulse in the input–output sequence and *m* represents the interval of the impulses prior to the present impulse within the kernel memory window *M*, *m* = 0 denotes the present input. The input to the system can be expressed as a series of variable-amplitude, random-interval delta functions:

(2) $$\begin{array}{c} x\left(t_{i}\right)=\sum_{i=1}^{I}A_{i}\delta \left(t-t_{i}\right) \end{array}$$

where *i* is the index number of impulses and *I* is the total number of impulses. The time of occurrence of the *i*^th^ impulse is *t*_*i*_. In the DG-CA1 trajectory model experiment, DG population spike amplitude were used as input (*A*_*i*_) and CA1 fEPSP amplitude were used as output *y(n)*. Because the input amplitude is varied, in order to isolate influence from present input, we considered the zero-lag terms in the original Volterra series (1) independently, as follows:

$$\begin{array}{l} y(n)=k_{0}+k_{1}(0)x(n)+\sum_{m=1}^{M}k_{1}(m)x(n-m)\\ \;+k_{2}(0,0)x(n)x(n)+\sum_{m_{1}=1}^{M}k_{2}(m_{1},0)x(n-m_{1})x(n)\\ \;+\sum_{m_{2}=1}^{M}k_{2}(0,m_{2})x(n)x(n-m_{2})\\ \;+\sum_{m_{1}\;=1}^{M}\sum_{m_{2}=1}^{M}k_{2}(m_{1},m_{2})x(n-m_{1})x(n-m_{2})+\ldots \end{array}$$

and can be then rearranged as:

(3) $$\begin{array}{c} \begin{array}{l} y(n)=k_{0}+k_{1}(0)x(n)+k_{2}(0,0)x{\left(n\right)}^{2}+\sum_{m=1}^{M}k_{1}(m)x(n-m)\\ \;+\sum_{m_{1}=1}^{M}\sum_{m_{2}=1}^{M}k_{2}(m_{1},m_{2})x(n-m_{1})x(n-m_{2})\\ \;+2\sum_{m=1}^{M}k_{2}(m)x(n)x(n-m)+\ldots \end{array} \end{array}$$

The first three terms on right represent the static input–output curve. The last three terms describe the nonlinear dynamical effect of the inputs on the output. In order to reduce the number of open parameters, an estimation of the kernels is facilitated by expanding them on the orthonormal Laguerre basis functions *L* (Marmarelis, 1993):

$$L_{l}(m)=\alpha^{(m\;-l)/2}{\left(1-\alpha \right)}^{1/2}\sum_{k=0}^{l}{\left(-1\right)}^{k}\left(\begin{array}{c} m\\ k \end{array}\right)\left(\begin{array}{c} l\\ k \end{array}\right)\alpha^{l-k}{\left(1-\alpha \right)}^{k}$$

where α is the Laguerre parameter (0 < α < 1) and affects the time extent of the basis functions. The convolution of Laguerre basis functions *L* and inputs *x* can be represented as

$$v_{l}(t_{i})=\sum_{t_{i}\;-\mu <t_{j}\;\leq t_{i}}A_{j}L_{l}(t_{i}-t_{j})$$

where *A*_*j*_ is the input spike amplitude in (2), *t*_*i*_ is the time of occurrence of the current impulse in the input–output sequence and *t*_*j*_ is the time of occurrence of the *j*^th^ impulse prior to the present impulse within the kernel memory window μ. The adapted Laguerre expansion of Volterra kernels with *L* basis functions can be rewritten as:

(4) $$\begin{array}{c} \begin{array}{l} y(t_{i})=c_{0}+A_{i}c_{1}(0)+A_{i}^{2}c_{2}(0,0)+\sum_{l=1}^{L}c_{1}(l)v_{l}(t_{i})\\ \;+\sum_{l_{1}=1}^{L}\sum_{l_{2}=1}^{L}c_{2}(l_{1},l_{2})v_{l_{1}}(t_{i})v_{l_{2}}(t_{i})\\ \;+2A_{i}\sum_{l=1}^{L}c_{2}(l)v_{l}(t_{i})+\ldots \end{array} \end{array}$$

where *c*_0_, *c*_1_, *c*_2_,… are the kernel expansion coefficients. Since the number of basis functions can be made much smaller than the memory length, the number of open parameters is greatly reduced by this expansion technique. The kernel expansion coefficients (*c*_0_, *c*_1_, *c*_2_,…) can be estimated via the least-squares method, and can be used to reconstruct the Volterra kernels (*k*_*i*_) using Laguerre basis functions (*L*)

$$k_{0}=c_{0},\;k_{1}=\sum_{l\;=1}^{L}c_{l}L_{l},\;k_{2}=\sum_{l_{i}=1}^{L}\sum_{l_{i}=1}^{L}c_{l_{1},l_{2}}L_{l_{1},l_{2}}$$

#### CA1 plant model implementation

***RARIT-induced monosynaptic data collection and analysis.*** After collecting the FARIT data for building DG-CA1 trajectory model, in the same slice, paired-pulse stimulation was applied to the *stratum radiatum* from a pair of stimulation electrodes in the conformal array in order to elicit the monosynaptic CA1 response. The pair of stimulation electrodes was selected according to their location and their ability to evoke typical paired-pulse facilitation. In this set of experiments, the amplitudes of the FARITs were modified to formulate a random-amplitude, random-interval trains (RARITs, Gaussian distributed, mean amplitude: 150 μA, which is the mean CA1 evoked postsynaptic potential amplitude observed in the FARIT-induced trisynaptic dataset). Once the pair of stimulation electrodes were determined, four 300-pulse RARITs were delivered to the slice. A channel from the CA1 region was selected and fEPSP amplitudes were analyzed for suitability in constructing the CA1 plant model.

***CA1 plant model configuration.*** The same Laguerre-Volterra (LV) modeling approach described in section “Trajectory Model Configuration” was applied to build a CA1 plant model. In this set of experiments, the amplitudes of the RARITs (from previous section) were used as measures of the input signal *A*_*i*_ in (2), and the fEPSP amplitudes of CA1 were used as measures of output. Once the input–output transformation of the CA1 system was fully explored, the inverse model can be further derived.

#### Inverse CA1 plant model implementation

***Inverse CA1 plant model configuration.*** The inverse model was built to transform the output (i.e., desired output of a CA1 region) to the input (i.e., desired input stimulation to a CA1 region). To develop the inverse model based on the LV model, the original Equation (4) was rearranged to:

(5) $$\begin{array}{c} \begin{array}{l} {}[c_{2}(0,0)]A_{i}^{2}+\left[c_{1}(0)+2\sum_{l=1}^{L}c_{2}(l)v_{l}(t_{i})\right]A_{i}\\ \;+[c_{0}+\sum_{l=1}^{L}c_{1}(l)v_{l}(t_{i})+\sum_{l_{1}=1}^{L}\sum_{l_{2}=1}^{L}c_{2}(l_{1},l_{2})v_{l_{1}}(t_{i})\\ \;\times v_{l_{2}}(t_{i})-y(t_{i})]=0 \end{array} \end{array}$$

In (5), the desired output *y* and the coefficients *c*_0_, *c*_1_, *c*_2_,… were obtained during process of model estimation. All the convolution terms could also be determined using the coefficients and previous stimulation amplitudes A_*i* − 1_, A_*i* − 2_,…. Once all the terms are determined, (5) became a quadratic equation with unknown desired input stimulation (*A*). It can be simplified as:

(6) $$\begin{array}{c} aA^{2}+bA+c=0 \end{array}$$

where

$$\begin{array}{l} a=c_{2}(0,0),\;b=c_{1}(0)+2\sum_{l=1}^{L}c_{2}(l)v_{l}(t_{i}),\;\\ c=c_{0}+\sum_{l=1}^{L}c_{1}(l)v_{l}(t_{i})+\sum_{l_{1}=1}^{L}\sum_{l_{2}=1}^{L}c_{2}(l_{1},l_{2})v_{l_{1}}(t_{i})v_{l_{2}}(t_{i})-y(t_{i}) \end{array}$$

such that the transformation of the inverse model (output to input) became an operation of solving *A* in (6). In this study, the roots of the quadratic equation can all be calculated from

(7) $$\begin{array}{c} A=\frac{-b+\sqrt{b^{2}-4ac}}{2a} \end{array}$$

The validation of this inverse model implementation is shown in the result (section “Inverse CA1 Plant Model Implementation and Validation Results”). All the calculated stimulation amplitude were used to recompose to the new stimulation trains, called desired-amplitude RITs (DARITs) as described below.

***DARIT-induced monosynaptic data collection and analysis.*** In this set of experiments, the amplitude of the RARITs (as used in section “RARIT-Induced Monosynaptic Data Collection and Analysis”) were reformed using the optimal stimulation amplitudes calculated from the inverse CA1 plant model (from previous section “Inverse CA1 Plant Model Configuration”), named DARITs. Four 300-pulse DARITs were delivered to the slice through the same pair of stimulation electrodes as RARIT experiments. A channel from the CA1 region was selected and the fEPSP amplitudes were analyzed.

#### Model validation

In this study, data were evaluated using the Variance Accounted For (VAF) and the Normalized Mean Square Error (NMSE) as described below:

$$\begin{array}{l} \;VAF=(1-\text{var}(Y_{i}-X_{i})/\text{var}(Y_{i}))\\ NMSE=\sum_{i}{\left(Y_{i}-X_{i}\right)}^{2}/\sum_{i}Y^{2} \end{array}$$

where *X* is the predicted amplitude of the model, *Y* is the amplitude analyzed from the recorded data, and *var* is the variance of the data. Specific data sets were chosen for comparison and are presented in the result section. To evaluate the prediction power of the models, we have used a cross-validation method, i.e., independent datasets are used for model estimation and model prediction. All model goodness-of-fit reported in this paper are obtained using this method.

### Modeling-control framework experiment protocol

**Figure 3:**
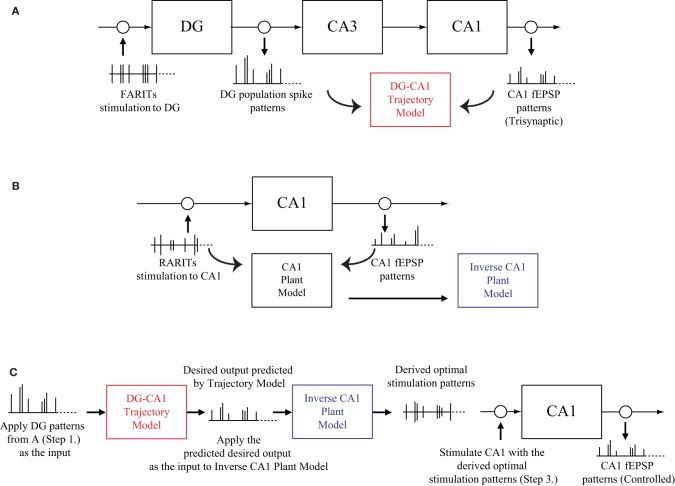
**A schematic diagram of the modeling-control experimental protocol. (A)** Stimulating the perforant path with FARITs, and analyzing the trisynaptic responses in DG and CA1, to build a DG-CA1 trajectory model. **(B)** Stimulating the Schaffer collaterals with RARITs, and analyzing the monosynaptic responses in CA1, to build a CA1 plant model. The inverse CA1 plant model can then be formulated. **(C)** Applying DG patterns (from **A**) as the input to DG-CA1 trajectory model (built in **A**) to predict desired CA1 output patterns; applying the predicted desired CA1 output as the input to the inverse CA1 plant model (built in **B**) to derive the optimal stimulation patterns; stimulating at the Schaffer collaterals with the derived optimal stimulation patterns, and then analyzing the responses in CA1. CA1 fEPSP amplitudes from **A** and **C** can then be compared.

The experiment protocol to verify our modeling-control paradigm for an *in vitro* hippocampal prosthesis involves following steps:

1. Stimulating the perforant path (DG input) with FARITs, and analyzing the trisynaptic responses in DG and CA1; applying DG population spike patterns as input and CA1 fEPSP patterns as output, building a DG-CA1 trajectory model using an LV kernel modeling approach (Figure 3A).
2. Stimulating the Schaffer collaterals (CA1 input) with RARITs, and analyzing the monosynaptic responses in CA1; applying RARITs patterns as input and CA1 fEPSP patterns as output, building a CA1 plant model using the LV kernel modeling approach, and then formulating an inverse CA1 plant model (Figure 3B).
3. Applying DG patterns as the input to DG-CA1 trajectory model to predict desired CA1 output patterns; applying the predicted desired CA1 output as the input to inverse CA1 plant model, to derive the optimal stimulation patterns; stimulating at the Schaffer collaterals with the derived optimal stimulation patterns, and then analyzing the responses in CA1 (Figure 3C).
4. Comparing the CA1 fEPSP amplitudes from Step 1 to those in Step 3.

## Results

The diagrams and performance of the DG-CA1 trajectory and CA1 plant model prediction, and the inverse CA1 plant model implementation are presented in this section. The presented protocol was conducted in six experiments. In each experiment, two sets of data were collected from a hippocampal slice. The first dataset was composed of the FARIT-induced trisynaptic data and was used to build the DG-CA1 trajectory model. The second dataset was composed of the RARIT-induced monosynaptic data and was used to build the CA1 plant model. The resulting two built models and their predictions are presented. This section also include the implementation of the inverse CA1 plant model, and lastly, the validation of the modeling-control paradigm used for regulating CA1 nonlinear dynamics.

### DG-CA1 trajectory model and the prediction results

**Figure 4:**
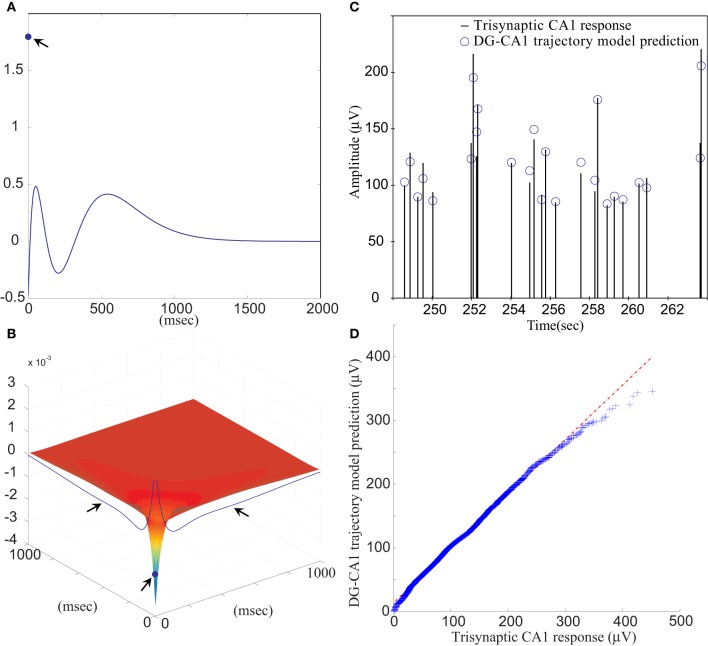
**(A)** The first order and **(B)** the second order LV kernel of the DG-CA1 trajectory model. The singular points and lines showing on the edge of each figure (indicated by arrows) represent the effect of present input. **(C)** A segment of comparison between a FARIT-induced trisynaptic CA1 response amplitude and the amplitude predicted by the DG-CA1 trajectory model. **(D)** The Q–Q plot of the data distribution between actual trisynaptic CA1 responses recorded from the slice and the outputs predicted by the DG-CA1 trajectory model.

The FARIT-induced hippocampal trisynaptic data were analyzed for use in building the DG-CA1 trajectory model. The amplitudes of evoked DG population spikes were used as measures of the input to the system, and the amplitudes of evoked CA1 fEPSPs were used as measures of output of the system. An LV kernel model was applied to study the nonlinearity of this system. Examples of the first and the second order LV kernels are shown in Figures 4A,B, respectively.

It should be noted that in Figure 4A, the singular point represents the *K*_1_(0) term in (3). The different polarity in Figure 4A manifests the importance of isolating the zero lag terms. In Figure 4B, the singular point represents the *K*_2_(0,0) term in (3), and the two lines indicated by arrows represent the ∑^*M*^_*m*_1_ = 1_∑^*M*^_*m*_2_ = 1_*k*_2_(*m*_1_, *m*_2_) term, while one of the input pairs is the present input (*m* = 0).

Model estimation was completed using population spike amplitudes and the intervals of the input–output sequences. From all datasets, the slice response amplitudes were analyzed and compared with the predicted amplitudes. The mean VAF was 65.97 ± 17.30%. In Figure 4C, a segment of FARIT-induced trisynaptic CA1 fEPSP amplitudes is compared to its counterpart predicted from the DG-CA1 trajectory model. The result is further confirmed by an overall comparison between the actual responses and model predicted outputs, as shown in Figure 4D. The quantile–quantile (Q–Q) plot demonstrates that the actual trisynaptic CA1 responses recorded from the slice are accurately predicted by the DG-CA1 trajectory model.

### CA1 plant model and the prediction results

**Figure 5:**
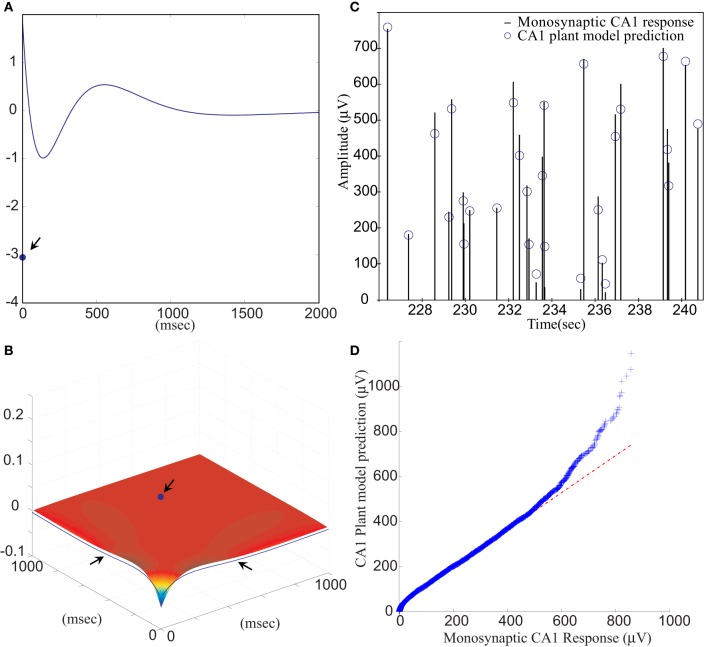
**(A)** The first order and **(B)** the second order LV kernel of the CA1 plant model. The singular points and lines showing on the edge of each figure (indicated by arrows) represent the effect of present input. **(C)** A segment of comparison between RARIT-induced monosynaptic CA1 response amplitudes and the amplitudes predicted by the CA1 plant model. **(D)** The Q–Q plot of the data distribution between actual monosynaptic CA1 responses recorded from the slice and the outputs predicted by the CA1 plant model.

The RARIT-induced CA1 monosynaptic data were analyzed for use in building the CA1 plant model. The random amplitudes of the RARITs were used as measures of input into the system, and the amplitudes of evoked CA1 fEPSPs were used as measures of the output of the system. The LV kernel model was applied to study the nonlinearity of the CA1 system. Examples of the first and the second order LV kernels are shown in Figures 5A,B, respectively.

Model estimation was completed using stimulation intensities and intervals of the input–output sequences. The VAF between slice response and model prediction was 85.56 ±13.91%, averaged from six datasets. This shows that the CA1 plant model can accurately predict CA1 amplitudes based on stimulation amplitudes. A segment of the RARIT-induced monosynaptic CA1 fEPSP amplitudes is compared to its counterpart predicted by the CA1 plant model, as shown in Figure 5C. Figure 5D displays the Q–Q plot of the overall monosynaptic CA1 responses and CA1 plant model predicted results.

### Inverse CA1 plant model implementation and validation results

The implementation of the inverse CA1 plant model is accomplished using RARIT-induced monosynaptic data. The purpose for formulating such an inverse model is to transform output predictions into input stimulations. The output predictions were acquired from the CA1 plant model, and applied as the *y* in (5). The coefficients *c*_0_, *c*_1_, *c*_2_,… were obtained during process of model estimation. Three terms involved the convolution of Laguerre basis functions and input amplitude were unknown, which include

$$\sum_{l=1}^{L}\text{​}c_{2}(l)v_{l}(t_{i}),\;\sum_{l=\;1}^{L}\text{​}c_{1}(l)v_{l}(t_{i}),\text{ and }\sum_{l_{1}=1}^{L}\sum_{l_{2}=1}^{L}c_{2}(l_{1},l_{2})v_{l_{1}}(t_{i})v_{l_{2}}(t_{i}).$$

Based on our experimental design, no stimulation existed before the stimulation train was sent, so these unknown terms were equal to zero. Thus, the first stimulation amplitude can be calculated by (7). After the first stimulation amplitude was calculated, it was then applied to convolve with the Laguerre basis function and formulate the unknown terms for calculating the next stimulation amplitude. The operations for solving the root were run through all data points in order to process the transformation from output into input. As a result, the inverse model allows us to convert the desired output response amplitudes to input stimulation amplitudes in a dynamic, recursive manner.

**Figure 6:**
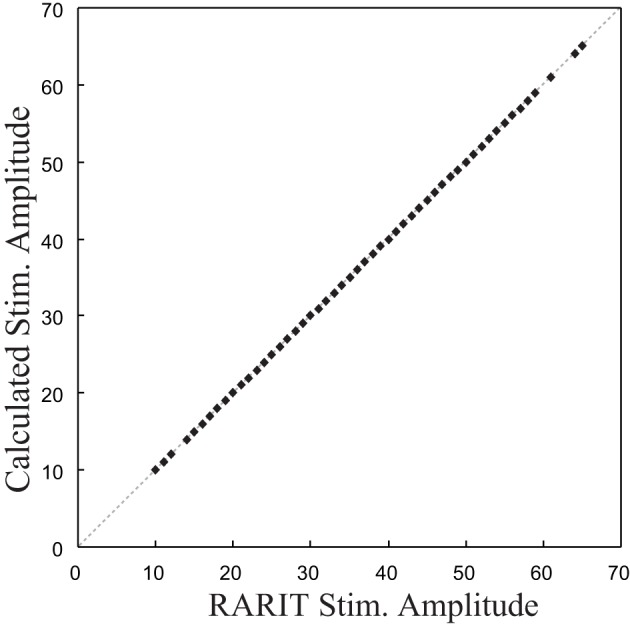
**The comparison between stimulation amplitude in RARIT that sent into CA1 and the amplitude calculated from inverse CA1 plant model**.

The validation of this inverse model was completed by comparing the calculated stimulation amplitudes with the RARIT amplitudes. The scatter plot in Figure 6 shows that the calculated stimulation amplitudes and the RARIT stimulation amplitudes are identical, showing that: (1) the real roots could all be calculated; and (2) this inverse model implementation can successfully derive optimal stimulations based on desired response amplitudes.

### Modeling-control results

**Figure 7:**
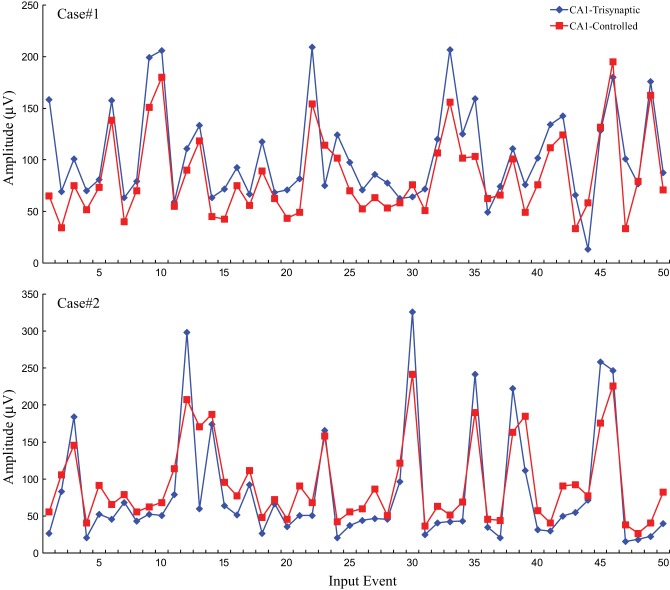
**A comparison of CA1 fEPSP amplitudes in response to FARIT-induced trisynaptic response (CA1-trysynaptic in blue diamonds) and DARIT-induced monosynaptic response (CA1-Controlled in red squares).** The NMSE from 6 datasets was 15.41 ±8.35%.

Following the protocol in this modeling-control framework experiment, CA1 desired output is first predicted through DG-CA1 trajectory model, and then applied into the inverse CA1 plant model to derive the optimal stimulation amplitudes. These amplitudes were used to formulate DARITs and were then sent into the slice, and the monosynaptic CA1 responses were recorded. The proposed modeling-control framework was intended to evoke CA1 to produce activities similar to the original CA1 activities. Thus, DARIT-induced monosynaptic CA1 amplitudes were compared with FARIT-induced trisynaptic CA1 response amplitudes. Two examples are shown in Figure 7.

**Figure 8:**
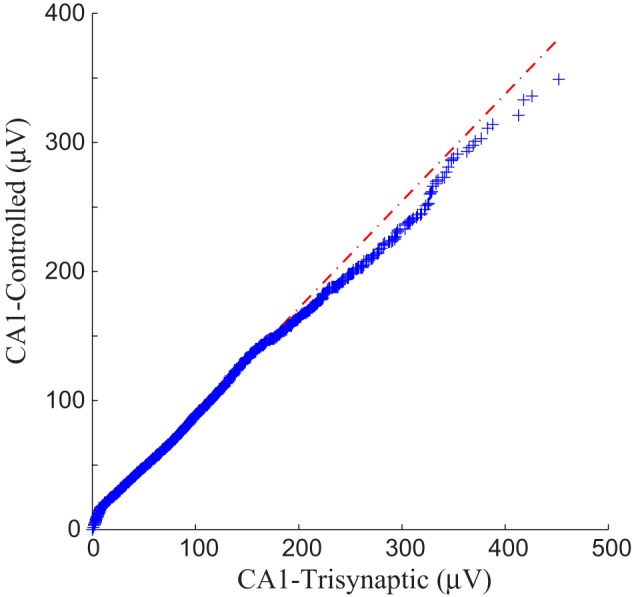
**A Q–Q plot demonstration of the data comparison between the trisynaptic CA1 responses and the paradigm controlled CA1 responses**.

Each panel illustrates results from one experiment: amplitudes of fEPSPs recorded from the CA1 region are shown as a function of 50 impulses chosen from among 2400 impulses of the stimulation trains (1200 administered with FARIT stimulation; 1200 administered with DARIT stimulation). In order to collapse the *x* axis to comprise more data points, time intervals between impulses are not represented in the figures; only “Input Event” number (sequence of sample impulses) is shown. Data for the FARIT-induced trisynaptic CA1 responses (CA1-trisynaptic) are shown in blue diamonds; data for the DARIT-induced monosynaptic CA1 responses (CA1-Controlled) are shown in red squares. As seen in Figure 7, the variation in CA1 fEPSP amplitudes was also captured in our model controlled paradigm. The accuracy was evaluated using NMSE of the amplitude, and the average NMSE was 15.41 ±8.35%. A Q–Q plot compared through the entire data sets is shown in Figure 8.

## Discussions

One of the essential objectives of a neural prosthetic device is to recreate the desired neural responses. While it is important to develop a reliable hardware model to represent the computational functions of a system, the control between device stimuli and actual responses is equally important. For example, in the application of deep brain stimulation (DBS), many efforts have been made in calibrating the stimulation parameters to achieve the desired effect (Mayberg et al., 2005; Okun et al., 2005). DBS devices depend on a trial-and-error process for finding the optimal stimulation pattern. Patients must repeatedly perform an exercise for a neurologist to adjust the stimulation parameters such as voltage, amplitude, pulse width, frequency, and electrode position (Moro et al., 2002; Volkmann et al., 2002; O'Suilleabhain et al., 2003). Another example is the application of functional electrical stimulation (FES) (Riener, 1999; Duffell et al., 2008; Donovan-Hall et al., 2011). The basic principle of FES is the generation of action potentials in the uninjured lower motor neurons by external electrical stimulation. This device faces problems such as muscle fatigue, spasticity, and limited force in the stimulated muscle. Using control strategies is one way to avoid internal disturbances and improve the time-consuming trial-and-error adjustment (Matjacic et al., 2003; Braz et al., 2009). Current neural prostheses face the same problem—the stimulation signals need to be adjusted manually or empirically based on the output response. In this article, we describe a rigorous approach to generate the stimulation patterns using a modeling-control framework. In the hippocampal slice preparation, with the purpose of restoring the CA1 output responses observed in the intact trisynaptic (DG to CA3 to CA1) circuitry, a nonlinear trajectory model was built to predict the CA1 desired output based on DG input. The predicted CA1 output was then converted to optimal stimulation through an inverse plant model of CA1 (i.e., an inverse transformation of CA1 input–output properties). Thus, the stimulation was essentially derived based on the desired output response, and was used to reactivate the CA1 response. An experimental validation of this modeling-control paradigm using hippocampal slices is provided. One of our preliminary studies was to stimulate CA1 region with non-optimal stimulation parameters, which means the nonlinearity of CA1 input–output relationship was not take into concern. The average NMSE from four experiments was 35.23 ±18.21%, which is much higher than the modeling-control paradigm results.

In current experimental paradigm, the only open parameters is the stimulation intensity, since the desired output responses are single EPSPs, the stimulations are the standard biphasic pulses, and there is no frequency (multiple impulses will elicit undesired multiple EPSPs). We are aware of the fact that in other applications (e.g., DBS), the phase and frequency are equally important parameters and also could be optimized. Our current modeling-control paradigm can be extended and used as a platform for the optimization of those parameters in the future. We understand that the stimulation site is also critical for this kind of devices, sometimes the misplacement of the electrode lead could cause poor efficacy or adverse effects (Richardson et al., 2009; van den Munckhof et al., 2010). Current clinical DBS surgeries were assisted with preoperative images analysis (MRI or CT), and intra-operatively guided with computerized stereotactic techniques. The lead could also be switched with limited-adjustability to compensate the inappropriate placement issue. By applying the stimulation electrode array in current experimental setup, the optimal stimulation site and its influence to the model can be further evaluated.

Our demonstrations also show that implementing a bidirectional neural prosthesis implicitly replaces the damaged system. In this approach, we do not need to explicitly estimate the transformational property of the CA3 region in the trisynaptic circuit. As long as we have the trajectory responses of the output system and once we identify the nonlinear input–output relationship of the output system, we are able to drive it to the desired output through its inverse model. One potential issue here would be “How to know the desired trajectory responses in the intact system?” In our opinion, there are several solutions/mechanisms that can mitigate this problem. First, we may develop a “generic model” from data recorded in normal animals. Previous studies have shown that there are significant amount of common features in the functional input–output properties across different animals, despite the animal-to-animal variations. In the case described in this study, all trajectory models are qualitatively similar in terms of the kernel polarity, kernel duration and kernel shape. Using model derived from other animals is imperfect, but at least provides a good approximation. Second, in behaving animal applications, the “imperfect” outputs generated by the “imperfect model” will be read out by the downstream brain regions. Neural plasticity, which is ubiquitous in the central nervous system, may play a role in adapting the system to the imperfect outputs or model. Third, more sophisticated computational methods such as reinforcement learning can potentially be used to develop self-adaptive or co-adaptive models.

**Figure 9:**
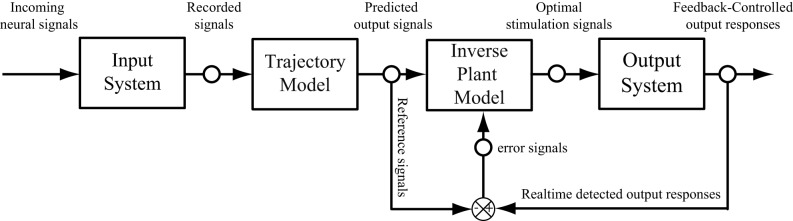
**A schematic diagram of a neuroprosthesis implemented with a feedforward and feedback controller**.

The paradigm introduced in this paper did not include the error feedback. This was based on the assumption that the error observed in the output responses is instantaneous and does not influence the future output. In order to extend the paradigm to a closed-loop feedback system (Bernotas et al., 1986; Houk, 1988; Veltink et al., 1992; Abbas and Riener, 2001; Liu et al., 2011), the output error, that may caused by the interface between electrodes and nervous systems, the variation of the system, or the internal disturbance, need to be considered. The trajectory model developed in this study can be used as a reference model, which provide the desired output responses to compare with the actual responses recorded from output system, to calculate the error signal (Figure 9). In this case, the feedback error will be used as an external input signal and sent to adjust the properties of the inverse model. The influence of the previous errors on the current output may be taken in to account in a dynamic manner. In such a scheme, the optimal stimulation signals are calculated by the inverse plant model based on both the input and error signals.

### Conflict of interest statement

The authors declare that the research was conducted in the absence of any commercial or financial relationships that could be construed as a potential conflict of interest.
